# Biomaterial-Based Schwann Cell Transplantation and Schwann Cell-Derived Biomaterials for Nerve Regeneration

**DOI:** 10.3389/fncel.2022.926222

**Published:** 2022-06-28

**Authors:** Zilong Rao, Zudong Lin, Panpan Song, Daping Quan, Ying Bai

**Affiliations:** ^1^Guangdong Engineering Technology Research Centre for Functional Biomaterials, School of Materials Science and Engineering, Sun Yat-sen University, Guangzhou, China; ^2^PCFM Lab, GD HPPC Lab, School of Chemistry, Sun Yat-sen University, Guangzhou, China

**Keywords:** Schwann cells, cell transplantation, biomaterials, peripheral nerve injury, central nerve injury

## Abstract

Schwann cells (SCs) dominate the regenerative behaviors after peripheral nerve injury by supporting axonal regrowth and remyelination. Previous reports also demonstrated that the existence of SCs is beneficial for nerve regeneration after traumatic injuries in central nervous system. Therefore, the transplantation of SCs/SC-like cells serves as a feasible cell therapy to reconstruct the microenvironment and promote nerve functional recovery for both peripheral and central nerve injury repair. However, direct cell transplantation often leads to low efficacy, due to injection induced cell damage and rapid loss in the circulatory system. In recent years, biomaterials have received great attention as functional carriers for effective cell transplantation. To better mimic the extracellular matrix (ECM), many biodegradable materials have been engineered with compositional and/or topological cues to maintain the biological properties of the SCs/SCs-like cells. In addition, ECM components or factors secreted by SCs also actively contribute to nerve regeneration. Such cell-free transplantation approaches may provide great promise in clinical translation. In this review, we first present the current bio-scaffolds engineered for SC transplantation and their achievement in animal models and clinical applications. To this end, we focus on the physical and biological properties of different biomaterials and highlight how these properties affect the biological behaviors of the SCs/SC-like cells. Second, the SC-derived biomaterials are also reviewed and discussed. Finally, the relationship between SCs and functional biomaterials is summarized, and the trends of their future development are predicted toward clinical applications.

## Introduction

Central nervous system (CNS) and peripheral nervous system (PNS) constitute the large neural network in the human body. The CNS, which includes brain and spinal cord, communicates with other organs/tissues through the PNS, perceives and processes information from external environment and maintains the biological functions of living bodies. Neurons and glial cells are the main cell types in the nervous systems. Neurons can receive internal and external stimuli, conduct nerve impulses, and integrate information. Most neurons have multiple dendrites and one axon. The axon conducts nerve impulses from the soma to the downstream neuron or effectors (such as myocytes). Meanwhile the dendrites receive information and transmit it to the soma. Glial cells, such as Schwann cells (SCs) in the PNS and oligodendrocytes in the CNS, wrap around axons and form insulating myelin structures, which facilitate rapid conduction of electrical impulses and protect neurons. Specific injuries in the nervous system (e.g., neurodegenerative disease, stroke, traumatic injury in the CNS, and peripheral nerve injury in the PNS) may disturb the interactions between neurons and glial cells, even result in complete impairment of the motor/sensory activities of the whole body.

### Peripheral Nerve Injury

Peripheral nerve injury (PNI), which is usually caused by mechanical trauma (e.g., traffic accidents, tool injuries) and disease complications (e.g., tumorectomy), often results in long-term numbness, loss of motor/sensory function, neuropathic pain, or paralysis (Schmidt and Leach, 2003). Based on the degree of damage, peripheral nerve injuries are classified into three categories: neurapraxia, axonotmesis, and neurotmesis (Sunderland, 1951). Neurapraxia and axonotmesis are usually caused by compression, overstretch, and nerve crush, which exhibit moderate symptoms and better prognosis compared to neurotmesis. The basal lamina around axons/SCs and the connective neuronal stroma (endoneurium, perineurium, and epineurium) is preserved after neurapraxia and axonotmesis, leaving an intact and permissive environment for axonal regeneration. Unfortunately, most PNIs found in clinical practice are neurotmesis or nerve transection, in which the continuity of the peripheral nerve is disrupted, and the connective tissues are partially or completely damaged (Barrette et al., 2008). Between the proximal and distal stumps of the injured nerves, the physical guidance and biochemical support are lost, which was originally provided by basal lamina tubes and connective tissues, resulting in disorientation of the regenerating axons. Therefore, the main objective of PNI treatments is to bridge the nerve stumps and help guiding severed axons to reach their disconnected targets (Siemionow et al., 2010).

The most frequently used clinical treatment of PNI is either direct suture of the severed ends or bridging the proximal and distal stumps using autologous nerve grafts. However, these two methods have distinct limitations and drawbacks. The former is only effective for PNI injuries with small gaps (<1 cm) (Bassilios Habre et al., 2018). For long-distance (>1 cm) gaps, transplantation of autologous nerve grafts achieves significant repair effect, which serves as the gold standard for PNI repair. Large amount of SCs and basal lamina tubes remain in the autologous nerve grafts that provide sufficient physical guidance and neurotrophic factors to promote axonal regeneration (Jiang et al., 2010). However, autologous nerve transplantation requires harvest of healthy nerves, resulting in loss of sensory and motor function at the donor site, along with possible neuromas and scar formation. Moreover, the transplanted nerves are usually sensory nerves that might lead to mismatch against the nerve size of the recipient region or bundle branches, which is why only about 50% of the patients reached effective functional recovery after autologous nerve transplantation (Daly et al., 2012; Sabongi et al., 2014).

Artificial nerve grafts, also termed nerve guidance conduits (NGCs), often serve as the alternatives to autologous nerve grafts. Many FDA approved biocompatible and biodegradable polymers are used for NGCs fabrication, such as poly (L-lactic acid) (PLLA), poly (ε-caprolactone) (PCL), silk fibroin, alginate, and the extracellular matrix (ECM) derived materials, including collagen, hyaluronic acid, and fibrin (Gu et al., 2014b). The NGCs can be prepared by filling in or coating on specific topological structures (e.g., microgrooves and aligned fibers) to support and guide axonal regrowth (Sarker et al., 2018a; Yang et al., 2021). However, these engineered nerve grafts only achieved similar or lower outcomes in repairing small nerve gaps (<3 cm) compared to the autografts, but the functional recovery was still unsatisfying for long-distance nerve defects. The most significant drawback of these NGCs is the lack of cellular support within the conduits. Transplantation of SCs is a feasible treatment to provide sufficient cellular components in the injured sites, and ultimately support axonal regeneration. Within this context, Guenard et al. (1992) transplanted SCs in a rat sciatic nerve defect, which demonstrated enhanced axonal regeneration and myelin formation. In addition, autologous SCs can be isolated from the patient and expanded *in vitro* prior to implantation, which serve as functional cell type for auto-transplantation therapies.

### Central Nerve Injury

The CNS possesses unique characteristics of structural and functional compositions compared to the PNS, and the pathological responses following central nerve injury (CNI) is also significantly different from PNI. The CNS is made up of gray matter and white matter, the gray matter mainly consists of neuronal somas, dendrites, and glial cells (astrocytes, microglia, and oligodendrocytes), and the white matter mainly consists of axons and glial cells (Assinck et al., 2017a). The gray matter forms a butterfly shape in the center of a spinal cord, which is wrapped around by the white matter or distributes on the periphery of the white matter in brain. Following CNI, large number of neurons and glial cells are dead and axons at the injury site are demyelinated. Unlike the PNS, myelin in the CNS is produced by oligodendrocytes rather than SCs. The myelin debris cannot be completely cleared after CNI due to apoptosis or silence of oligodendrocytes (Barres et al., 1993). The residual myelin debris and other inhibitory molecules further lead to inflammatory responses, including astrocyte proliferation and glial scar formation, resulting in a non-permissive microenvironment that prohibits neural stem cell differentiation into neurons and axonal regeneration (Cao et al., 2001; Pallini et al., 2005).

Facing with such a complex pathology after CNI, the treatment strategies usually require overcoming the specific pathological factors in the inhibitory microenvironment, which should reconstruct a favorable microenvironment for axonal regeneration (Pego et al., 2012). Therefore, current research on CNI treatment include blocking the inhibitory factors against axonal growth, such as Nogo-A, MAG, and CSPG that produced by myelin debris, or replacing ECM to activate the regeneration capacity of endogenous neurons. Anti-inflammatory drugs, such as minocycline, IL-10, indomethacin, and many neurotrophic factors, including nerve growth factor (NGF), neurotrophin-3 (NT-3), and brain-derived neurotrophic factor (BDNF) are also employed to alleviate inflammatory responses, promote neuron survival, and facilitate axonal regeneration (Cao et al., 2001; Sahni and Kessler, 2010). However, these strategies are mainly neuroprotective, aiming to prevent secondary cell death, minimize the extent of injury, or enhance neural plasticity by targeting only one inhibitory substance. Although their potential has been demonstrated by previous research, these single-target strategies cannot overcome the complex barriers toward regeneration, limiting their efficacy in promoting functional recovery. Therefore, a combinational approach is desired to resolve multiple issues after CNI.

Cell transplantation is considered to potentially ameliorate the injured environment in CNI treatments. The SCs and SC-like cells are the most promising cell lineages for cell-based therapies. As a cytokine bank in the PNS, SCs produce a variety of growth factors, including NGF, BDNF, and glial cell line-derived neurotrophic factors (GDNF), as well as some ECM proteins, such as laminin and fibronectin, which can stimulate the intrinsic capability of the damaged neurons to survive and facilitate axonal extension (Bixby et al., 1988). More importantly, considering that SCs can participate in myelin debris clearance and promote remyelination after PNI, which may provide viable solutions to those intractable problems caused by inactivity of oligodendrocytes after CNI. Furthermore, it was reported that SCs transplantation induced axon regeneration from retinal ganglion cells (RGCs) and peripheral-type myelination in a rat optic nerve injury model (Li et al., 2007). Therefore, SCs transplantation is potentially multi-functional for facilitating axonal regeneration and myelination in the CNI treatments (Hill et al., 2006).

### The Role of Schwann Cells in Nerve Regeneration

**Figure 1:**
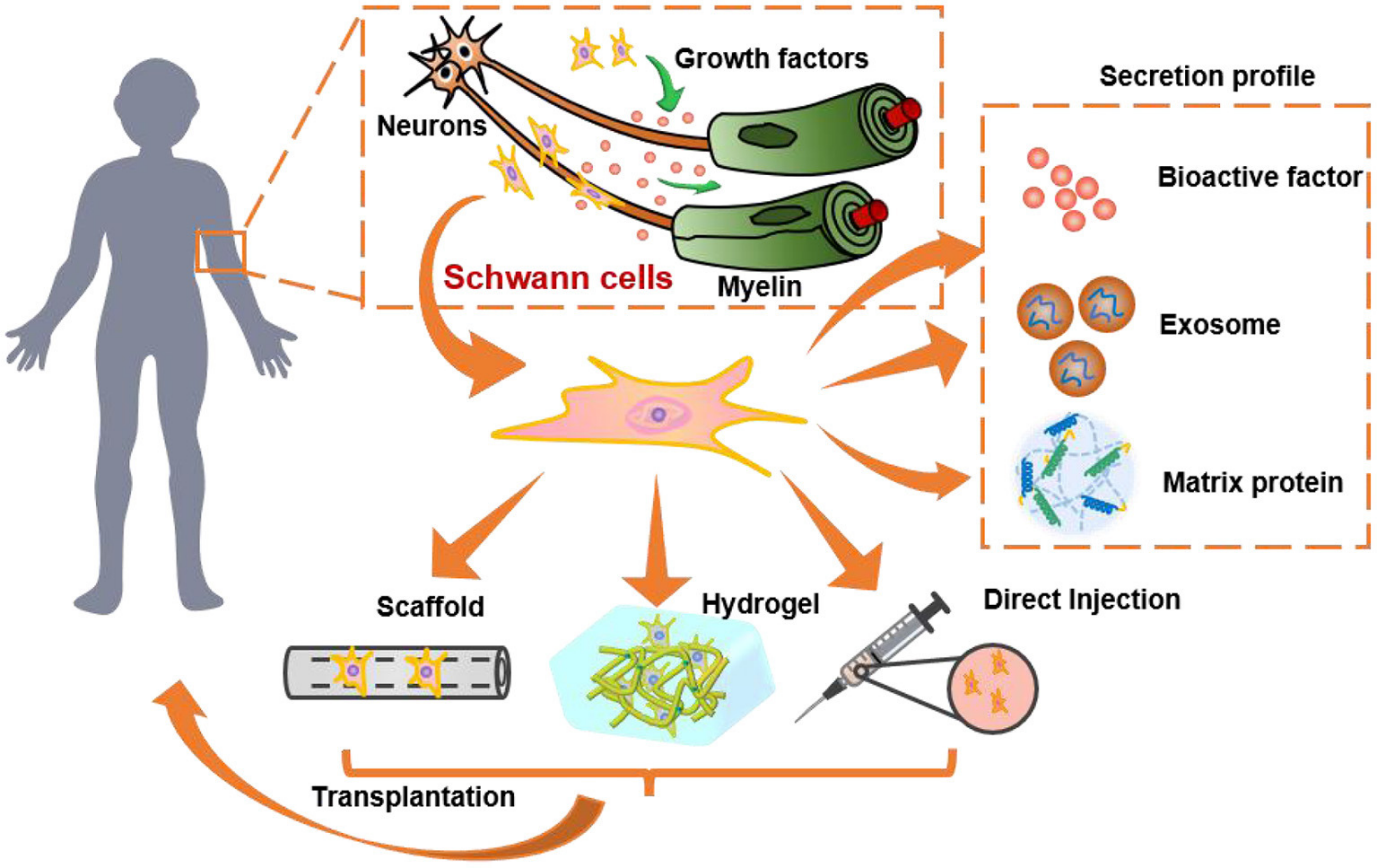
The role and of SCs and SC-derived materials in nervous system and their transplantation for nerve regeneration. SCs secret growth factors to protect neurons, promote neurite growth, and wrap around axons to form myelin. They can be transplanted by direct injection into humans or pre-encapsulated in 3D hydrogels/scaffolds for transplantation. Besides, the secretion profile of SCs, such as the bioactive factors, exosomes, and extracellular matrix proteins, are also promising alternatives to SCs transplantation.

After nerve injury, series of cellular and molecular changes occur at the injury site, including the changes of interaction between extracellular matrix and neural cells, which are potentially related to nerve regeneration (Gaudin et al., 2016). SCs play a pivotal role in several aspects of nerve regeneration, such as nerve fiber regeneration, myelination, and axonal guidance (Figure 1). For example, the damaged axons degenerate, and myelin sheath disintegrates after PNI (Touma et al., 2007), SCs lose contact with axons and result in radical changes of the signaling environment. Subsequently, SCs are activated and shift from a mature myelinating/non-myelinating state to a proliferating and repair phenotype, which is known as the plasticity of SCs (Ma and Svaren, 2018; Jessen and Mirsky, 2019). Along with macrophages, the dedifferentiated SCs participate in removal of cellular debris and myelination through autophagy (Zhao and Yi, 2019). This unique characteristic of SCs provides strong regeneration capacity in peripheral nerves. On the contrary, residual myelin debris usually remains uncleared at the injury site that leads to inhibitory microenvironment after CNI.

Once the myelin debris is cleared, series of regeneration-supporting neurotrophic factors and growth-inducing molecules, such as GDNF, BDNF, NGF, vascular endothelial growth factor (VEGF), and N-cadherin are secreted by SCs to promote neuron survival and axonal elongation (Ziegler et al., 2011; Wood and Mackinnon, 2015). The expression profile of different factors follows a temporal sequence that the protective factors (e.g., GDNF, NGF) are secreted earlier than the growth promoting factors (e.g., BDNF) (Gomez-Sanchez et al., 2017). Simultaneously, the dedifferentiated SCs begin to proliferate, elongate, and align within the residual endoneurial tube to form Büngner bands, which provides a growth pathway for the regenerating axons (Menorca et al., 2013; Sarker et al., 2018b). Following axonal regeneration, SCs wrap around the newborn axons to reconstruct myelin sheaths. This effect is considerably valuable for the treatments of both PNI and CNI, since the remyelination of regenerated axons is key to functional recovery of the injured nerves. Although SCs do not exist endogenously in the CNS, previous studies verified that some non-myelinating and myelinating SCs can be recruited from the PNS to promote myelin repair after CNI (Ma et al., 2018). Moreover, oligodendrocyte precursor cells (OPCs) in the CNS may differentiate into remyelinating SCs in response to demyelination and injury (Assinck et al., 2017b). These results strongly support the capacity of SCs to contribute in CNI repair.

Currently, the treatments that rely on the endogenous SCs are quite unsatisfactory, particularly for the long-distance peripheral nerve lesions. Although SCs are sensitive to environmental signals because of their plasticity, they cannot sustain their repair phenotype in long-term due to the gradually changing signals. Notably, it was evident that senescence-related SC markers (e.g., *SA-*β*gal*) is up-regulated in long-distance nerve injury and key regulators of the repair phenotype (e.g., *C-Jun*) is delayed, resulting in diminishing repairing effect (Saheb-Al-Zamani et al., 2013; Poppler et al., 2016; Chen et al., 2017). The transplantation of SCs may serve as an effective cell-based approach for the treatments of nerve injury.

### Schwann Cells Transplantation for Nerve Injury Repair

Although the pathological responses in the PNS and CNS are quite different after injury, their repair strategies still share some common features. For example, researchers employed exogenous implants to improve the microenvironment of the injured area, stimulate the regenerative capacity of endogenous neural stem cells, guide axonal extension, and promote remyelination of the regenerated nerves. In recent years, numerous studies have been conducted using different therapeutic strategies, such as drugs, growth factors, and biomaterial scaffolds, for nerve injury repair (Gaudin et al., 2016; Sarker et al., 2018a). Although positive outcomes have been evident, the repair effects are still limited, especially for those severe nervous system injuries. The reason is that it is difficult to mimic the complex biochemical and physical characteristics of the native tissues, moreover, a long-lasting and favorable microenvironment is critical for nerve regeneration.

Cell-based therapies, especially SCs transplantation, are promising for the treatments of both PNI and CNI. As the most common glial cells in peripheral nerves, SCs are involved in the construction of extracellular matrix during peripheral nerve development, which can also actively participate in promoting nerve regeneration after PNI and CNI. However, there are two important issues that need to be addressed in SCs transplantation. First, the source of SCs is limited. Although the current technology allows extraction and purification of autologous SCs, it is difficult to obtain sufficient quantity and good quality of SCs for effective transplantation. The second issue is that the injection-based transplantation usually leads to significant shear-induced cell death. Furthermore, the injected cells are often rapidly lost at the injected region, resulting in unsatisfied survival *in situ*. On the other hand, most of the transplanted cells cannot transform into their regenerative phenotype spontaneously, but easily affected by the negative environmental factors and lose their regeneration capability at the injury site. Therefore, proper biomaterial-based cell carriers are highly desirable to address these two problems for the application prospects of SCs transplantation.

## Biomaterial-Assisted SCs Transplantation

### Transplantation of SCs and Schwann Cell-Like Cells

As the gold standard in treatments of peripheral nerve defects, transplantation of nerve autografts is the main approach to introduce autogenous SCs to the injury site after PNI, but the disadvantages of autografts are well-noticed as previously described. Meanwhile, scaffold implantation is only applicable to nerve lesions with gaps (El Seblani et al., 2020). Purified SCs can be obtained by *in vitro* culture and expansion techniques, which is potentially beneficial for exogenous SCs transplantation. The *in-situ* injection of autogenous SCs cause less damage to the injury sites and is more suitable for irregular nerve defects or demyelinating diseases. The cultured SCs also allow biological/chemical SCs induction to achieve a regenerative phenotype before implantation. However, the quality and repair capacity of the patient derived SCs may be affected by their physiological conditions, such as the age of donors (Painter et al., 2014; Bastidas et al., 2017). Moreover, SCs isolation requires surgical removal of healthy nerves and long-term cultivation to render adequate cell number for transplantation. Therefore, alternative resources of SCs are desirable for clinical applications.

Schwann cell-like cells (SCLCs) derived/differentiated from stem cells provide a great promise as the SCs substitution for nerve injury repair (Hopf et al., 2020). Numerous stem cell sources, such as embryonic stem cells (ESCs), adult stem cells, and induced pluripotent stem cells (iPSCs), can differentiate into SCLCs though biological/chemical inductions (Dezawa et al., 2001; Huang et al., 2017; Kim et al., 2017). Ziegler et al. obtained SCLCs with SC-like morphological and molecular properties through induced differentiation from human ESCs, they demonstrated that these SCLCs interacted with axons and triggered myelination (Ziegler et al., 2011). Adult mesenchymal stem cell (MSCs), such as bone marrow stem cells (BMSCs) and adipose-derived stem cells (ADSCs), are also widely used in nerve injury therapies due to their high proliferation capability and multilineage differentiation potential (Park et al., 2010; Cai et al., 2017). Despite the capacity of direct differentiation into SCLCs, MSCs have also been identified to secret neurotrophic factors, cytokines, and immunomodulatory factors to support axonal growth and mediate immune response at the injury sites (Wang et al., 2009; Lin et al., 2012). MSCs secrete a variety of soluble factors, which include tumor necrosis factors (e.g., TNF-β1) and interleukins (e.g., IL-13 and IL-10) to exert anti-inflammatory potential and inhibit release of pro-inflammatory factors (such as TNF and IL-6), when transplanted into injured spinal cord (Sobacchi et al., 2017). Skin-derived precursors (SKPs) are another reliable source of SCLCs, since they originate from neural crest. SKP-differentiated SCLCs were identified to exhibit identical transcriptional signatures as in the primary SCs (Stratton et al., 2017). Unfortunately, these stem cells derived SCLCs have not yet progressed into clinical trials, because of heterogeneity, quality, and ethical restriction issues. SCLCs differentiated from iPSCs are currently more attractive, which may overcome the resource limitations of the ESCs and MSCs (Maherali et al., 2007). The iPSCs can be differentiated to neural crest cells and subsequently transformed into different neural cell lineages.

SCs and SCLCs can be delivered to the injured nerves through different ways. The simplest method is to prepare a suspension of the SCs and SCLCs in medium and then directly inject into the injury sites (Pinero et al., 2018; Mousavi et al., 2019; Assinck et al., 2020). Tomita et al. transdifferentiated ADSCs into SC phenotype, the cell suspension was then slowly injected into the distal region of the reconnected nerves. Compared to the allogenic SCs, ADSCs-derived SCs exhibited comparable capability in facilitating nerve regeneration after chronic denervation (Tomita et al., 2012). However, direct cell injection often has several serious drawbacks. First, the shear stress during needle-based microinjection causes irreversible damage to the cells. Second, the transplanted cells may disperse rapidly and lose their repair capacity due to the unfavorable microenvironment *in situ*. Especially for the stem cell-derived SCLCs, the SC-like phenotype is usually unstable and easily transformed into other cell types. The direct injection approach is also limited since the injury sites must be enclosed. Therefore, it is probably more applicable to the neurogenic diseases in brain, or injuries caused by contusions in spinal cord or peripheral nerve. To ensure high therapeutic efficacy after SCs or SCLCs transplantation, proper cell carriers are highly desired to reduce shear damage and provide physical and biological support for cell growth, retention, and nerve regeneration.

### SCs Transplantation Using Injectable Hydrogels

To avoid severe damage to the cells during injection, the SCs or SCLCs can be pre-suspended in pre-gel solution for *in-situ* gelation or pre-encapsulated in hydrogel. Hydrogel is a hydrated polymer network with high water content (Drury and Mooney, 2003). It can provide a three-dimensional environment and similar structural/mechanical properties as the native tissues, thus has received considerable attentions in tissue engineering (Liu et al., 2020). Before gelation, the hydrogels present a liquid-like state (i.e., pre-gel solution), while cells can be mixed and encapsulated in the pre-gel solution. The polymer chains in the hydrogel can protect the cells from rupture and provide shear resistance during injection, resulting in promoted survival and viability of the transplanted cells. Following injection, the cells containing pre-gel solution can be induced to form hydrogel in response to temperature change or by exposure to light. The high porosity and permeability of the hydrogels allow the exchange of oxygen, nutrients, and cytokines with surrounding tissues to support cell survival and growth (Szabo et al., 2019).

In general, the hydrogels used for SCs or SCLSs transplantation need to meet some essential requirements. For instance, hydrogels must be biocompatible and non-toxic to the cells and host tissues. The pre-gel solution should exhibit low viscosity and gel quickly when delivered to the injured area, ensuring a rapid settlement and retention of the transplanted cells (Marquardt and Heilshorn, 2016). The implanted hydrogels are desired to resist compression and infiltration of the surrounding tissues, while providing suitable stiffness that allow attachment and functionalization of the SCs/SCLCs. Therefore, injectable hydrogel with mechanical strength ranging from 100 Pa to 10 kPa are often chosen to match with that of the native nerve tissue (Budday et al., 2017) and they are required to have adaptable stability to prevent degradation-induced loss of the transplanted cells. On the other hand, as regeneration progresses, hydrogels gradually degrade to allow impending tissue regrowth, and finally replaced by the ECM secreted by the surrounding cells, such as neurons and glial cells. Besides the physical properties, bioactivities that promotes revascularization are also desirable using hydrogels as transplantation carriers. It has been reported that the implanted cells can survive surrounded by blood vessels, otherwise they would be hard to obtain nutritional support. Therefore, rapid revascularization within hydrogels plays an important role in the survival of cell transplantation (Daly et al., 2020). Therefore, choosing a proper hydrogel with suitable mechanical and biological properties significantly influence the subsequent *in vivo* performance of the transplanted SCs/SCLCs for tissue repair.

Injectable hydrogels can be classified as natural and synthetic hydrogels, based on their origins (Table 1). Natural hydrogels derived from the ECM components in animals and plants are popular choices for SCs transplantation, due to their resemblance to the native nerves (Geckil et al., 2010; Li and Lepski, 2013). The most widely used natural hydrogels include proteins (e.g., collagen and fibrin) and polysaccharides (e.g., alginate and hyaluronic acid) (Gyles et al., 2017). They possess intrinsic amino acids or peptide domains that bind with cell receptors and typically contribute to cell adhesion and axonal growth in the native tissues, which make them biocompatible and bioactive in modulation of cellular functions (Catoira et al., 2019). Therefore, natural hydrogels have great advantages to serve as cell carriers, since they provide favorable environment for the encapsulated cells and support their survival and proliferation.

**Table 1:** Hydrogels used for SCs/SCLCs transplantation.

	**Biomaterial**	**Origin**	**Advantages**	**Disadvantages**	**Application**
Protein-based hydrogel	Collagen	Animal tissues, a structural protein of the ECM	Easy to be isolated, purified, and can spontaneously form hydrogels Biocompatible and biodegradable Provide essential ECM molecules to support cellular functions Nanofibrous structures	Potential immunoreaction Inadequate mechanical property Rapid degradation *in vivo*	The raw material of FDA-approved nerve conduits Promote cellular survival, retention, and neurite regeneration as a cell carrier (Guan et al., 2013; Georgiou et al., 2015)
	Gelatin/GelMA	A product of hydrolyzed collagen	Good biocompatibility, degradability, low immunogenicity, and stable mechanical properties.	Potential damage to the transplanted cells during UV light crosslinking	The base of bio-ink in 3D bioprinting and cell encapsulation (Zhao et al., 2016; Sun et al., 2018; Xiao et al., 2019).
	Fibrin	An insoluble protein polymer of plasmic proteins	Highly compatible with blood and tissues Inhibit the expression of myelin and keep SCs in a non-myelin state to induce regeneration through ERK1/2 phosphorylation	Poor stiffness, fast degradation, and the risk of transmitting blood disease.	Enhanced SCs viability and nerve regeneration by introducing fibrin hydrogel (Schuh et al.) Promote EMSC differentiation into SCLCs (Chen et al., 2015).
Polysaccharides-based hydrogel	HA	Animal tissues, rich in eyes and joints, a glycosaminoglycan type of ECM content	Biocompatible and biodegradable Easy to be chemical functionalized Promote cell proliferation by binding to CD44 receptor on cell surface Shear-thinning	Poor cell attachment property due to the high-water retention rate	Combined with alginate hydrogel to promote SCs survival and proliferation (Wang et al., 2013) Provide injectability, printability, and bioactivity to protect SCs and SCLCs during transplantation (May et al., 2018; Ho et al., 2019)
	Alginate	A linear polysaccharide extracted from brown algae	Non-immunogenicity, slow degradation properties and high hydrophilicity Quickly and reversibly crosslinked by Ca^2+^	Need biological modification	Bridging materials for both spinal cord and peripheral nerve injury repair (Mosahebi et al., 2002; Wu et al., 2020).
Decellularized matrix-based hydrogel	Tissue-derived dECM hydrogels	The native mammal tissues/organs	Retain large amount of ECM components, and recapitulate the biological characteristics of native ECM Biocompatible, biodegradable, and bioactive	Lower moduli, difficult to be modified Unknown immunoreaction in human	Peripheral nerve-derived dECM hydrogel significantly improved SCs survival and axonal remyelination (Cerqueira et al., 2018)
	Matrigel	Cultured EHS tumor cell lines	Excellent biological activity in promoting cell growth and proliferation.	Potential risk in clinical translation	Base material for cell culture (Kamada et al., 2005; Lopatina et al., 2011).
Synthetic hydrogels	PEG		Non-toxicity, good biocompatibility, low protein adsorption, and noninflammatory invasion. Reduces ROS to protect neurons.	Hydrophilic and biological inert Undegradable	Modified with RGD peptides or other functional peptides to support NSCs and SCs survival (Franco et al., 2011; Marquardt et al., 2020)
	PHEMA		Porosity and moduli are easily manipulated	Bio-inert	Supporting materials of the multicomponent hydrogels (Hejcl et al., 2010)

#### Protein-Based Hydrogel

As the major protein components of the ECM in nerve tissues, collagen type I is one of the most commonly used natural biomaterials for nerve regeneration. Collagen can be easily isolated, purified, and spontaneously form hydrogel at temperature ~37°C through adjusting the pH value (Silva et al., 2021). Collagen hydrogel not only provides essential ECM molecules to support cellular functions, but also mimics the nanofibrous structure of native nerves, which is beneficial for cell adhesion and proliferation. Marchand et al. injected a pre-cooled collagen I solution into a transected spinal cord injury rat model, the collagen self-assembled *in situ* and formed nanofibrous hydrogel. Eight days post-surgery, the host cells, such as fibroblasts, macrophages, and SCs were observed to penetrate into the hydrogel, and the regenerated axons were found crossing the injury boundary into the hydrogel after 3 months (Marchand and Woerly, 1990). Considering the positive cellular response, collagen has been widely used as the raw material in nerve conduits, as well as a popular cell carrier for SCs and SCLCs. It has been proven that when MSCs were transplanted using collagen gel, the survival and retention of the MSCs were increased compared to injection of MSCs alone (Guan et al., 2013). Georgiou et al. encapsulated both rat SCs and ADSCs-derived SCs (dADSCs) into collagen and induced cell alignment by plastic compression. The collagen supported the secretion of functional molecules and growth factors from the SCs and dADSCs, which guided neurite growth *in vitro*, and promoted nerve regeneration in a 15-mm rat sciatic nerve defect model cpmp (Georgiou et al., 2015). Due to the processability of hydrogel, collagen gel can be employed in many fabrication technologies, including lyophilization, 3D printing, and electrospinning, to expand its the clinical applicability. However, since collagen is derived from animal tissues, the implantation of collagen in human raises safety concerns that may cause immunoreaction (Friess, 1998).

Gelatin is a product of hydrolyzed collagen, which retains the amino acid sequences of collagen, such as the classic arginine-glycine-aspartate (RGD) peptide. Gelatin possesses good biocompatibility, degradability, and low immunogenicity, which supports cell infiltration, adhesion, and proliferation (Echave et al., 2017). However, gelatin becomes soluble at body temperature but gels at lower temperature, which makes it unsuitable for *in-situ* transplantation. To overcome this drawback, gelatin is frequently modified by crosslinking agents, or incorporate with other materials to ensure a stable state during implantation. Methacrylated gelatin (GelMA), produced by substituting amines in gelatin with methacrylamide, can form photocrosslinkable hydrogel with additional photoinitiator. Due to the rapid gelation and stable mechanical properties, GelMA has been broadly used for 3D bioprinting and cell encapsulation (Zhao et al., 2016; Sun et al., 2018; Xiao et al., 2019). Luo et al. encapsulated dental pulp stem cells (DPSCs) and bFGF in GelMA hydrogel to repair 15-mm long sciatic nerve defect in rats, which achieved functional recovery that comparable to nerve autograft (Luo et al., 2022). Wang et al. fabricated a bio-ink by mixing 10% GelMA solution with rat SCs and BMSCs, then underwent extrusion-based bioprinting to construct a bionic spinal cord stent. By changing the cellular components in the GelMA-based bioink, different spatial arrangement of SCs and BMSCs was achieved that facilitated motor function recovery after spinal cord injury (Wang et al., 2021).

Fibrin is derived from plasmic proteins and highly compatible with blood and tissues, which has received extensive attention in the field of wound healing, due to its capability in clotting and hemostatic properties (Mosesson, 2005). Moreover, fibrin also plays a special role in peripheral nerve regeneration. Following PNI, the blood-nerve barrier broke down and fibrinogen plasma can leak into the injured area, and then be cleaved by thrombin to form fibrin cables. The fibrin cables not only serve as physical guidance for SCs migration, but also interact with SCs to induce their regenerative phenotype by upregulating p75NGFR through ERK1/2 phosphorylation (Akassoglou et al., 2002; Petersen et al., 2018). Therefore, fibrin hydrogel can be viewed as a potential transplantation platform for SCs. Schuh et al. blended fibrin with collagen to form an engineered neural tissue, where fibrin was used as an activator for the transplanted SCs. It was confirmed that the addition of fibrin enabled significant enhancement of SCs viability and promoted nerve growth both *in vitro* and *in vivo*, compared to collagen (Schuh et al., 2018). Chen et al. confirmed that ectomesenchymal stem cells (EMSCs) grown on the fibrin hydrogel expressed myelination-related molecules such as myelin basic protein (MBP) and galactocerebrosides (GalCer), and secreted more neurotrophins compared to those grown on a plastic surface. They further demonstrated that fibrin hydrogel promoted EMSC differentiation into SCLCs (Chen et al., 2015). However, it should also be considered that the poor stiffness, fast degradation, and the risk of transmitting blood disease may limit the clinical applications of fibrin hydrogels.

#### Polysaccharides-Based Hydrogel

Polysaccharides are a sort of biopolymers consisting of monosaccharides or disaccharides, which can also be found easily in ECM (Russo et al., 2015). Polysaccharides play important roles in the composition of cell membranes, intercellular communication, and energy storage of living organisms in animals and plants. For example, hyaluronic acid (HA) is a linear, highly hydrated, non-sulfated anionic glycosaminoglycan (GAG) polymer consisting of β-1,4-D-glucuronide and β-1,3-N-acetylglucosamine. It is one of the most abundant components in the ECM of nervous tissues, which is involved in the regulation of many cellular functions, such as cell adhesion, proliferation, migration/maintenance of the connective tissue, and proteoglycan assembly (Zamboni et al., 2018). HA can specifically bind to CD44 receptor on cell surface and activate intracellular signaling pathways, such as the Ras-Raf-MEK-ERK pathway, to trigger cell proliferation (Ponta et al., 2003; Matsumoto et al., 2009). However, HA cannot be crosslinked directly and often exhibit poor cell attachment properties. Therefore, chemical modification to introduce functional groups onto HA chains, such as methacrylate or thiol groups, is required to endow gel formation capabilities. HA hydrogel can be further mixed with other bioactive materials or undergo RGD peptide modification to promote cell adhesion. A hyaluronan/methylcellulose (HAMC)-based hydrogel was proven to be a versatile cell delivery system, which improved the survival and distribution of transplanted retinal stem cells (RSCs) and neural stem cells (NSCs) in stroke and spinal cord injury models (Ho et al., 2019). The HA component of the HAMC hydrogel is shear-thinning to endow injectability and exhibited enhanced tissue response, while the methylcellulose component underwent inversed thermal-gelling and formed physical crosslinking. May et al. encapsulated skin-derived precursor Schwann cells (SKP-SCs) in the HAMC hydrogel, and the survival of grafted SKP-SCs was improved when injected into spinal cord cavity. The modification by laminin and fibronectin further reduced cell spreading effect and anoikis compared to the HAMC hydrogel alone (May et al., 2018).

Alginate is a negative-charge linear polysaccharide extracted from the cell wall and intercellular mucilage of brown algae. Alginate consists of α-guluronic acid and β-D-mannuronic acid that can rapidly crosslink with multivalent cations to form an ionotropic hydrogel (Sun and Tan, 2013). In the past decade, alginate hydrogel has been widely used as bridging materials for both spinal cord and peripheral nerve injury repair. It can host exogenous SCs, stem cells, and neurotrophic factors. Mosahebi et al. transplanted allogeneic SCs pre-embedded in alginate hydrogel into a 10-mm sciatic nerve injury model. The SCs exhibited repair functions to promote sustained axonal regeneration compared to the alginate hydrogel without SCs (Mosahebi et al., 2002). Alginate hydrogel usually exhibits non-immunogenicity and slow degradation properties. However, some research studies reported that alginate prevents cell adhesion due to its high hydrophilicity, presents bioinert properties for nerve regeneration, or even inhibits neurite outgrowth (Novikova et al., 2006). Therefore, biological modifications are necessary for the application of alginate hydrogel as a platform for SCs transplantation. For example, Wu et al. formed a bioprinted 3D scaffold using a composite bio-ink consisting of gelatin and alginate with the presence of SCs, the content of gelatin facilitated SCs adhesion to the scaffold (Wu et al., 2020).

#### Decellularized Matrix-Based Hydrogel

Due to the complexity of organisms, single component biomaterials, such as proteins and polysaccharides, can only partially recapitulate the compositional and biological characteristics of the native ECM. ECM consists of a variety of proteins, proteoglycans, and glycosaminoglycans, which is secreted and constructed by the resident cells of tissues or organs, providing biological and topographical cues for cell growth (Hussey et al., 2018). In recent decades, decellularization have brought an alternative option to obtain ECM-mimicking biomaterials with tissue-specific properties by removing the cellular components from the native mammal tissues/organs. Compared to the single component materials, decellularized extracellular matrix (dECM) retains large amount of ECM components from natural tissues, including proteins, polysaccharides, and growth factors, which exhibit high biological activities. Collagen is the major component of ECM and mostly preserved in the dECM, which further enables the digested dECM to form nanofibrous hydrogel though self-assembly. These dECM hydrogels are highly biocompatible that may better recapitulate the 3D microenvironment in native tissues, which also can serve as promising cargos for cell transplantation.

Among the various dECM hydrogels, Matrigel has been commercialized and widely used as a base material for cell culture. Matrigel is a cell-derived dECM hydrogels extracted from cultured Englebreth-Holm-Swarm (EHS) tumor cell lines by proteolytic digestion (Hughes et al., 2010). Matrigel contains most of the components of the basement membrane (~60% laminin, 30% IV collagen and 8% nestin, and cytokines, such as TGF-β, epidermal growth factor, and insulin-like growth factor) and shows excellent biological activity in promoting cell growth and proliferation. Therefore, Matrigel may serve as a good vehicle for the transplantation of SCs and stem cells (Kamada et al., 2005; Lopatina et al., 2011). Patel et al. encapsulated SCs into three different matrices, methylcellulose, mixture of laminin and collagen IV, and Matrigel, respectively. After transplantation into rat spinal cords, the SCs encapsulated within Matrigel enhanced SCs survival and facilitated angiogenesis and axonal in-growth (Patel et al., 2010). Williams et al. used Matrigel/allogenic SCs transplantation to treat transected spinal cord injury in rats. They found that the implantation of fluid Matrigel and SCs improved regeneration of brainstem axons across the rostral interface, compared to the pre-gelled Matrigel (Williams et al., 2015). Although Matrigel exhibits great biological activity as a 3D culture platform for many different cell types, its tumor-secreted nature leads to extremely high risk in clinical translation.

Unlike Matrigel, dECMs that harvested from animal tissues/organs and decellularized by chemical/physical methods are more clinically acceptable, which have become a hot topic of tissue engineering in recent years. The dECM scaffold can be enzymatically digested to obtain a decellularized matrix solution, and then neutralized to a physiological pH and gel at 37°C (Brightman et al., 2000). Particularly, similar to the natural environment of SCs, dECM hydrogel derived from peripheral nerves may serve as an excellent biomaterial carrier for SC transplantation. The bioactive molecules retained in the dECM hydrogel can synergistically maintain the viability and biological functions of the SCs. However, the utilization of nerve-derived dECM hydrogel on SCs or stem cell transplantation has been rarely reported. Cerqueira et al. (2018) used rat allogeneic peripheral nerve-derived dECM hydrogel (iPN) for SC transplantation into a rat spinal cord contusion model. They showed that the iPN significantly improved the survival of SCs after transplantation compared to Matrigel, and further promoted axonal myelination. On the other hand, the dECM hydrogels derived from various tissues, such as cardiac, liver, skin, and nerves have also shown great promise in cell delivery and regulation of cellular behaviors (Park et al., 2021; Xu et al., 2021). It is believed that the dECM hydrogels derived from the nervous system may serve as an optimal choice for SCs transplantation (Lin et al., 2018; Zou et al., 2018; Liu et al., 2021; Xu et al., 2021).

#### Synthetic Hydrogels

Natural hydrogels have shown their advantages in biocompatibility and bioactivity, but most natural hydrogels have relatively lower moduli ranging 100–1,000 Pa, which hardly resist the extrusion of surrounding tissues. The degradation of natural hydrogels is relatively fast due to the enzymatic environment *in vivo* and difficult to support a long-term nerve regeneration. Chemical-/photo-crosslinking is often employed to enhance the stiffness of natural hydrogels, but the modification of natural materials is difficult and might be harmful to their biological activities (Sionkowska, 2011). Synthetic polymers with biocompatibility and suitable biodegradability can also be used as cell carriers. The physical properties of the synthetic hydrogels, such as porosity, modulus, and degradation rate are easily manipulated. However, most of the synthetic materials lack bioactivity or specific binding sites for cellular interactions, making them unsuitable for cell adhesion or tissue attachment. To address these problems, the bioactivity of synthetic hydrogels is often introduced by immobilization of bioactive molecules, peptides, or blending with the natural polymers (Woerly et al., 2001).

Polyethylene glycol (PEG) based hydrogels are one of the most frequently used carriers for cell delivery due to its non-toxicity, biocompatibility, low protein adsorption, and non-inflammatory invasion (Pasut et al., 2016). More importantly, studies have shown that PEG can promote fusion of cell membranes and repair damaged membranes, which also reduces reactive oxygen species (ROS) to protect neurons (Shi, 2013). Once modified with acrylate, the resulting polyethylene glycol diacrylate (PEGDA) undergoes gelation under UV light. However, PEG hydrogel is usually highly hydrophilic and biological inert that is insufficient for cell adhesion, which makes PEG hydrogel alone unsuitable for cell transplantation. The bioactivity of PEG-based hydrogels can be improved through copolymerization with other polymers, modification with bioactive molecules, or blending with natural hydrogels. Franco et al. modified PEGDA with RGD peptides, and then microencapsulated neural stem cells within the PEG-RGD microspheres to repair a rat stroke model (Franco et al., 2011). Marquardt et al. designed a “SHIELD” hydrogel consisting of an 8-arm PEG polymer modified with proline-rich peptides, PNIPAM, and a recombinant engineering protein C7. This complex hydrogel matrix resulted in significant retention of the transplanted SCs compared to direct cell injection, and further improved functional recovery of spinal cord injury (Marquardt et al., 2020). Despite the nervous system, PEG-based hydrogels are extensively applied in encapsulating various cell types, such as islet cells, ESCs, and MSCs (Koffler et al., 2019).

There are also some other synthetic hydrogels that can potentially be used in SCs or SCLCs transplantation, such as poly (2-hydroxyethyl methacrylate) (PHEMA) and poly[N-(2-hydroxypropyl)-methacrylamide] (PHPMA) (Hejcl et al., 2010). Limited by the poor biological activities, synthetic hydrogels are not commonly used alone as cell carriers, but act as the supporting materials of the multicomponent hydrogels.

Overall, injectable hydrogels possess unique advantages in cell transplantation, since they can provide native-like 3D microenvironments that allow the transplanted cells to grow and proliferate (Alvarado-Velez et al., 2014). Meanwhile, cell transplantation using hydrogel-assisted injection is minimally invasive to patients and more clinically acceptable in the treatments of some neurodegenerative diseases.

### SCs Transplantation Using Scaffolds

The injectable hydrogels only provide biological cues for the survival and functionalization of SCs, while topological cues in native nerves that guide SCs alignment and polarization are missing. The alignment of SCs plays a critical role in nerve regeneration, since endogenic SCs are induced to align by the residual endoneurium tubes or the fibrous matrix, forming the Büngner bands to guide neurite growth. However, introducing physical cues to the hydrogels is quite difficult, since SCs or SCLCs are often randomly distributed inside the bulk hydrogels. In addition to the hydrogels, cells can also be transplanted using preformed scaffolds, including the nerve guidance conduits. The good mechanical property and processability of synthetic polymers, such as PEG, poly (lactic acid) (PLA), polycaprolactone (PCL), and poly-3-hydroxybutyrate (PHB) play an important role in scaffold preparation for SC transplantation. Bio-scaffolds can be fabricated with different architectures, such as the aligned fibers and microchannels, to regulate cellular behaviors, sometimes more suitable for the treatments of nerve defects. The limitation of biomaterial scaffolds is that the transplanted SCs often grow only on the surface of the scaffolds, which is quite different from the 3D hydrogels.

#### Scaffolds Without Topological Cues

Many scaffolds that have been used for transplanting SCs and SCLCs were simply fabricated with isotropic architectures, such as nanofibrous or interconnected porous structures. These scaffolds are either fabricated by solvent evaporation from hydrogels or polymer solutions using lyophilization or vacuum drying. Polymer solutions can be dispersed in a defined mold, then dehydrated into a tubular shape. The medium suspensions of SCs and SCLCs are infused into the lumen of the dry tube, and the tube can absorb the medium and distend to form a hydrogel-like or hydrated scaffold. For example, a gelatin hydrogel tube was first made by dehydration, followed by injection of SCs or ADSCs suspension. After incubation in the medium, the hydrated gelatin tube was implanted into a 5 mm-sciatic nerve lesion. The results confirmed that SCs were retained in the gelatin tube after 8 weeks (Sowa et al., 2016). In fact, a significant number of the hydrogel-based grafts are used as the cargo for SCs transplantation. Berrocal et al. implanted an absorbable collagen based NeuraGen® tube that combined with autologous SCs for treatment of a critical sized gap (13 mm) in the sciatic nerve of Fischer rat (Berrocal et al., 2013). The transplanted SCs survived at least for 4 months post-surgery and significantly enhanced the regeneration of myelinated axons.

The scaffold-assisted cell transplantation allows the combination of robust synthetic polymers with bioactive natural hydrogels. For example, PCL is a biodegradable polymer that has low degradation rate and good processability. Babaloo et al. (2019) fabricated a PCL/gelatin nanofibrous scaffold and co-seeded with human SCs (hSCs) and human endometrial stem cells (hEnSCs). The cell-laden PCL/gelatin scaffold was then transplanted into hemisected spinal cord. The co-cultured hEnSCs and hSCs successfully triggered hEnSCs differentiation into neuron-like cells and resulted in a better performance for SCI repair. Basically, the SCs are transplanted by adhering to the surface and the sponge-like pores of the scaffold, the microstructure of the scaffold may perform a positive impact on the cellular behaviors. Uz et al. (2017) constructed the 3D gelatin conduits with three different microstructures by thermally induced phase separation (TIPS). The results indicated that the gelatin conduits with microporous and ladder-like structures were beneficial for MSCs adhesion and proliferation, and the ladder-like structures were more favorable for MSCs differentiation into SC-like phenotypes than the microporous structures.

#### Scaffolds With Topological Cues

Given the tubular microstructure of the native nerve tissues and the directional connection between neurons and target tissues, anisotropic physical structures are crucial for SCs polarization, directed migration, and myelin formation. Introducing the oriented structures into the transplantation scaffolds allows the alignment of SCs, and the aligned SCs provide a direct pathway for neurite extension (Wang et al., 2015). For example, a significant difference was found when SCs were cultured on the aligned and randomly oriented poly (methyl methacrylate) (PMMA) nanofibers. When cultured on the aligned PMMA nanofibers, the SCs polarized and exhibited larger aspect ratio than those cultured on the non-aligned nanofibers, which were more likely to co-localize with neurites for further myelination (Xia et al., 2016). Electrospinning is a commonly used technique to prepare scaffolds with highly oriented nanofibrous structures. Lee et al. fabricated a polyvinylidene fluoride trifluoroethylene (PVDF-TrFE) membrane with aligned electrospun nanofibers, then rolled into a hollow conduit. By injection of SC-loaded Matrigel, the aligned PVDF-TrFE nanofibers were proven to be beneficial for SCs alignment *in vivo* and promoted axonal extension, compared to the randomly oriented nanofibers (Lee et al., 2017). Notably, electrospinning can hardly be used to process complex 3D architectures and the scaffolds are usually formed by rolling the electrospun meshes, which may diminish their orientation effect on the transplanted cells.

Unidirectional freeze-drying (UFD) is a unique approach to introduce longitudinally oriented microchannel structures inside the scaffold (Sridharan et al., 2015). Compared to the tubular conduits, scaffolds with 3D oriented structures prepared by UFD may possess larger specific surface area that allow SCs attachment. The resulting microchannels have been proven to guide SCs migration and neurite extension. Since the UFD technique utilizes the sublimation of unidirectional finger-like ice crystals to form microchannels, it is more applicable for the hydrogel-based natural materials, such as collagen, chitosan, and hyaluronic acid. Boecker et al. engineered a collagen conduit by UFD to transplant olfactory ensheathing cells (OECs), which is a type of glial cells that ensheaths olfactory axons to form myelin, similar to the SCs (Boecker et al., 2018). The suspension of OECs was perfused into the conduit and the cells aligned along the longitudinally oriented microchannels following conduit implantation. The OECs-containing conduits revealed local beneficial effects on axonal densities and myelination when compared to the empty scaffolds. Primary rat SCs were also transplanted with a UFD-prepared oriented collagen-chitosan conduit, which were observed to adhere and extend longitudinally along the microchannels (Zhang et al., 2013). The SCs-containing scaffolds closely resembled the microstructure and biological components of the native nerves, and provided a permissive microenvironment to promote axonal outgrowth and nerve regeneration.

In clinical practice, decellularized peripheral nerves are viewed as the most promising scaffolds for SCs transplantation (Han et al., 2019). Though the decellularized nerve grafts retain the longitudinally aligned tubular structures and most of the dECM compositions, the cellular components are removed from the native peripheral nerves, making them less effective in nerve regeneration compared to the autografts. The recellularization of SCs in the decellularized nerve grafts even more closely recapitulates the structure and bioactivity of the nerve autografts. Previous studies suggested that the decellularized nerves might support neuroregeneration in <30-mm peripheral nerve defects in non-human primates. Furthermore, Hess et al. (2007) combined the decellularized grafts with cultured autologous SCs which promoted remarkable regeneration in 6-cm ulnar nerve defects of macaca fascicularis. The decellularized allografts also provide pro-regenerative microenvironment for stem cells and induce their differentiation into SCLCs. Qiao et al. verified the SC-like differentiation of dental pulp stem cells (DPSCs) when cultured on a xenogeneic acellular nerve graft (ANG), which was further transplanted to repair 10-mm sciatic nerve defect in rat (Qiao et al., 2019). Immunohistochemical and electrophysiological assessments confirmed that the addition of DPSCs resulted in better functional recovery compared with the ANG scaffold. Recently, a study investigated the efficiency of introducing human ADSCs into commercialized human decellularized nerve grafts (Avance®), which further promoted the clinical application of SCs transplantation (Mathot et al., 2020). Although using decellularized nerves as the SCs carriers is promising, the limited source and potential risk of disease transmission still urges the development of advanced artificial implants for SCs transplantation.

#### Introducing Topological Cues Into Hydrogels

The main purpose of introducing oriented microstructures into the implanted scaffolds is to induce SCs polarization and alignment. However, the SCs or SCLCs usually attach to the surface of the aligned fibers/tubes, indicating that the cells are pre-cultivated bi-dimensionally. Following implantation, the cells need to transfer from surface to a 3D environment consisting of body fluids and ECM. With the rapid development of fabrication technologies, research studies have been focusing on introduction of oriented structures into hydrogels, in which the transplanted cells can grow three dimensionally. A representative approach was reported by B. Phillips et al. who incorporated different cell types, including SCs, ADCSs, and DPSCs into collagen gel, respectively. The cell alignment was induced by plastic compression (Georgiou et al., 2013; Sanen et al., 2017; Schuh et al., 2018). They also fabricated an anisotropic SC-containing collagen hydrogel by a gel aspiration-ejection (GAE) system (Muangsanit et al., 2020). Through slowly aspirating the collagen hydrogel, the shear force conferred elongation and alignment of the SCs within the hydrogels. It was also noted that the alignment of SCs maintained for long periods, induced that the number of regenerated axons and extent of vascularization was superior to the empty conduits in a rat sciatic nerve injury model.

Bioprinting shows great potential in producing cell-encapsulated hydrogel scaffolds for tissue engineering, which allows manipulation of spatial arrangement/distribution of the transplanting cells. Ning et al. modified a low viscosity alginate using RGD peptide, then blended with HA and fibrin to form the stock solution, and rat primary SCs were also incorporated to generate a functional bioink (Ning et al., 2019). They found that nearly 80% of the bioprinted SCs were aligned along the axial direction when the moving speed of the printing nozzle was 9 mm/s. Wu et al. printed a 3D conduit with SCs-containing gelatin and sodium alginate hydrogel, resulting in upregulated secretion of some growth factors from the SCs, including NGF, BDNF, and GDNF. Besides the alignment of SCs, the spatial distribution of SCs within the scaffold is important since their cellular functions usually rely on the accurate cell positioning. Bi-printing provides unique advantages in precise design and controlling the cellular distribution in the 3D scaffolds to mimic the native tissues or to meet special requirements. Wang et al. encapsulated BMSCs and SCs in two different GelMA-based bioinks, then bioprinted 3D scaffolds with spatial arrangement of the cells, respectively (Wang et al., 2021). Compared to the homogeneous hydrogel encapsulated with both cells, the 3D-printed scaffold with spatial distribution resulted in better functional recovery after spinal cord injury.

Interestingly, the transplanted cells can also be controlled and re-arranged into pre-designed patterns by external stimulation, such as the introduction of electromagnetic fields. Acoustic stimulation can promote cell migration via the density differences between cells and the surrounding matrix, which has been employed to fabricate complex cell patterns either on 2D plate or in 3D hydrogel. Using a heptagon acoustic tweezer, Gesellchen et al. created a SCs-linear pattern on a cover slip, and dorsal root ganglion (DRG) neurites were proven to grow along the SCs (Gesellchen et al., 2014). Although have been applied in 3D SCs alignment, the acoustic stimulation was used to form neuro-progenitor cells (NPCs) 3D construct in a fibrin hydrogel, which showed a great potential in cell transplantation therapy (Bouyer et al., 2016). Integrating magnetic-responsive short electrospun fibers into the hydrogel is another promising method to obtain cellular alignment in 3D environment. Prior to electrospinning, superparamagnetic iron oxide nanoparticles (SPIONs) were mixed into the polymer solution. The obtained electrospun nanofibers were cut into short fibers, then incorporated into the hydrogel. In response to the magnetic stimulation, the short fibers spontaneously arranged along the direction of the magnetic field, and further provided an oriented guidance for DRG neurites (Johnson et al., 2019). These abovementioned techniques achieved the distribution of cells without changing the chemical and mechanical properties of the implanted scaffolds. More importantly, the induced cell patterns were formed within the hydrogel, which allowed 3D cell growth. The external stimulation can be introduced either pre or post transplantation. The cell transplantation using injectable hydrogels with pre-aligned cells may meet various clinical demands, including minimal invasive surgeries and other features to repair long-distance nerve defects.

## Schwann Cell-Derived Biomaterials

Nerve injury results in dysfunction of the innervated organs, which is hardly recovered unless proper surgical treatments are performed. Since SCs can support axonal regeneration, transplantation of SCs has been proven to be effective in nerve injury repair. Despite that cell transplantation has been proven repeatedly by animal experiments, clinical applications of cell therapy are scarce, due to the potential host rejection and risk of tumorigenesis (Cai and Huang, 2006). In fact, intercellular communication relies on the secretion of cells, such as cytokines, exosomes, and ECM, which can serve as the modulators in regulating cellular behaviors through paracrine pathways. It has been reported that the components of SCs secreted factors can actively support axonal maintenance and regeneration, some of which have already been employed in bioscaffolds (Gordon, 2009). Growth factors are cell-secreted proteins or peptides that can regulate numerous physiological processes, such as differentiation, migration, adhesion, and gene expression. The importance of SCs-derived growth factors (including NGF, GDNF and BDNF) in nerve injury repair has been well-established and reviewed and discussed, as well as the strategy of assembling these growth factors within the engineered nerve grafts (Deister and Schmidt, 2006). In this review, we emphasize on the properties of the other two types of secretions, exosomes and ECM proteins, which are discussed from the application prospect as SCs-derived biomaterials for nerve injury repair.

### Transplantation of SCs-Derived Exosomes

Exosomes are nanoscale extracellular vesicles secreted by mammalian cells with a flattened spherical shape and diameters ranging from 30 to 150 nm (Thery et al., 2009). This nano-sized property of exosomes enables easy delivery through blood or other biological fluids. They also involve in many biological activities, such as immune regulation, metabolism, anti-oxidative stress, and tissue regeneration. Exosomes contain various functional proteins, mRNA, and micro-RNA (miRNA). Research studies have shown that exosomes can serve as an effective alternative to their origin cells in immunotherapy and regenerative medicine (Record et al., 2011). For example, glial cells in nervous systems secret exosomes with different functionalities. Lopez-Verrilli et al. (2013) first revealed that exosomes derived from SCs can be endocytosed by DRG axons, to promote the regrowth of transected DRG axons was significantly enhanced by continuous supplement of the SCs-derived exosomes. By direct injection into the injury site of a rat crushed nerve injury model, the introduced SCs-derived exosomes significantly increased the rate of axonal regeneration, indicating their potential in nerve injury repair. Subsequent studies showed that the SCs exosomes can inhibit GTPase RhoA activity and modulate growth cone dynamics to promote axonal regeneration, which may be beneficial in delaying cell senescence and apoptosis through inhibiting the caspase-3 signaling pathway (Lopez-Verrilli et al., 2013). Proteomic analysis revealed that several pathways related to microenvironment regulation were enriched by SCs-derived exosomes, such as neurotrophin secretion and PI3K-Akt signaling pathway (Wei et al., 2019). The miRNAs and proteins in SC-derived exosomes can regulate axonal regeneration and enhance neuron survival, which may play a critical role in potential therapeutic applications against nervous system diseases.

Although some common components such as tetraspanin (CD63, CD81, and CD9) and heat shock proteins (Hsp70, Hsp90) are involved, different cell phenotypes secrete different exosomes that carry various types of biological substances (Tamkovich et al., 2016). Lopez-Leal et al. found that only the exosomes secreted by the SCs of repair phenotype can facilitate axonal regeneration after nerve injury, rather than those exosomes secreted by the differentiated SCs (i.e., the mature and myelinating SCs) (Lopez-Leal et al., 2020). Interestingly, although the exosomes of differentiated SCs failed in promoting axonal growth, further studies demonstrated that they may inhibit SCs migration through secreting specific miRNAs and promote myelinization (Sohn et al., 2020). This discovery strongly supports the hypothesis that exosomes can recapitulate the specific features of their donor cells. However, the functions of SCs-derived exosomes have been investigated for nerve regeneration only in recent years, and the components and features of such exosomes remain unclear. Considering the neuroprotective and neuroregeneration effects, the SCs-derived exosomes have become a promising biomaterial that may serve as SCs substitute for transplantation therapies and nerve regeneration. Compared to SCs or SCLCs, their secreted exosomes provide several advantages, such as convenient storage, easy modification, and low risk of tumorigenesis, which make them even more suitable for tissue engineering applications.

The exosome-based therapies have been progressing rapidly in recent years, but the application of SCs-derived exosomes is rarely reported. The commonly used transplantation strategy was to resuspend SCs-derived exosomes in culture medium or saline solution, then introduced to the injured area through direct injection. Wang et al. continuously infused SCs-derived exosomes through tail vein in mice every 2 weeks to treat diabetic peripheral neuropathy (DPN) within 8 weeks (Wang et al., 2020). They confirmed that the SCs-derived exosomes can improve the neurological function in DPN mice and increase the amount of intraepidermal nerve fibers by supplementing specific miRNAs (miR-21,−27a, and−146a). However, direct exosome injection limits their therapeutic effects due to the rapid clearance *in vivo*, and a sustained delivery system using hydrogels or scaffolds is highly desirable to maintain their stability and biological functions. In a recent study, Matrigel was used to first encapsulate SKP-SCs-derived exosomes and then perfused into a silicon tube to fabricate the artificial nerve graft (Yu et al., 2021). The release profile showed that the exosome encapsulated Matrigel underwent sustained release for at least 8 weeks. Although satisfactory results were achieved in sciatic nerve regeneration, the Matrigel-based transplantation is extremely risky. The potential application of various other bioactive materials may serve as the exosome carriers, such as collagen and fibrin, and their synergistic effects need to be explored extensively in nerve injury repair.

### SCs-Derived Decellularized Extracellular Matrix

The ECM constitutes intrinsic physical and biochemical cues that can regulate cellular behavior and biological function in tissue development and regeneration. The composition and structure of ECM are determined by the type and function of the resident cells, which can be modified in response to various changes in the microenvironment, such as mechanical stimuli and nutrient concentrations. On the other side, ECM provides significant impact on the behaviors of the resident cells and maintains the biological functions of tissues/organs (Hussey et al., 2018). As the most important glial cells in peripheral nerves, ECM secreted from SCs contains a variety of macromolecules, such as collagens, laminins, and proteoglycans, during the development and regeneration process. These ECM components have been reported to be functional in inducing SCs alignment and proliferation. Therefore, materials extracted from the native ECM of peripheral nerves may serve as a promising alternative of SCs can be used as cell-free scaffolds for nerve regeneration (Khaing and Schmidt, 2012). Compared with individual ECM component, the compositional dECM reconstructs the 3D extracellular microenvironment that has been widely used in regenerative medicine. The dECM scaffolds derived from nerve tissues not only provide good platforms for SCs transplantation, but also possess great potential as the cell-free nerve grafts, due to their remarkable bioactivity and biocompatibility. During the past decades, decellularized nerve matrix has already been commercialized (“Avance,” Axogen, USA and “hANGs,” Guangzhou Zhongda Medical Device Company, China) for clinical applications, which becomes the most popular substitution of the nerve autografts. In the treatments of CNI, decellularized nerve grafts derived from peripheral nerves are also used. For example, Villegas-Perez et al. (1988) transplanted the decellularized nerve grafts to bridge the optic nerve defects and achieved a certain degree of tissue regeneration.

Furthermore, the hydrogels derived from the decellularized nerves also exhibit promising advantages in SCs regulation and nerve injury repair. Liu et al. (2021) prepared a porcine decellularized peripheral nerve matrix hydrogel, which exhibited high bioactivity in promoting axonal extension, SCs alignment, and nerve fiber remyelination. A consecutive study showed that the decellularized nerve matrix hydrogel coated electrospun PLLA nanofibers significantly promoted SCs migration and axonal extension (Chen et al., 2019; Deng et al., 2022). The synergistic effects of the decellularized nerve matrix hydrogel and the aligned nanofibers were further confirmed *in vivo* regarding nerve fiber fasciculation, myelination, and functionalization (Zheng et al., 2021). The capacity of decellularized nerve matrix hydrogel was also assessed by Lin et al. *via* perfusion into a nerve guidance conduit and implantation into a rat 15-mm sciatic nerve injury model (Lin et al., 2018). Rao et al. further induced longitudinally oriented microchannel structures in the decellularized nerve matrix hydrogel by UFD. They showed that the introduction of topological guidance promoted neurite extension and SCs migration both *in vitro* and *in vivo* (Rao et al., 2021). The decellularized nerve matrix hydrogel with aligned topology resulted in functional recovery comparable to those using nerve autografts. These results indicate that the decellularized matrix derived hydrogel holds great potential in clinical translations.

Although the decellularized nerve matrix and its derivatives are beneficial in promoting nerve regeneration, some drawbacks still exist, including source tissue scarcity, host responses, and potential pathogen transfer through heterogenic or allogenic transplantation (Badylak et al., 2009). Alternatively, the cell-derived decellularized dECMs, especially those extracted from autogenic cells, often possess low immunogenicity and are free of pathogenic factors transfer. Particularly, SCs-derived dECM is more refined than the decellularized nerve matrix, which recapitulates the functions of native SCs (Lu et al., 2011). In consequence, the dECM scaffolds derived from cultured SCs have been used as bioactive materials for nerve regeneration. Gu et al. (2014a) cultured primary rat SCs for 14 days and then underwent decellularization. Fibronectin and laminin preserved in the SCs-derived dECM, was used to seed DRG neurons which sprouted much longer axons than those cultured on poly-lysine coated plates. The SCs were further cultured on a chitosan/silk fibroin conduit and decellularized to obtain dECM modified nerve conduit, which resulted in repair effect that was comparable to the decellularized nerves in 10-mm rat sciatic nerve injury. Considering the difficulty in acquiring adequate autologous SCs, the dECM derived from stem cell differentiated SCs (such as the SKP-SCs) can also be used in scaffold modification (Zhu et al., 2018). However, the cell-derived dECMs are barely used directly due to the relatively low yields of ECM secretion, which often serve as the bioactive supplements. Although production issues remain unsolved, such as the control of costs, batch differences, and large-scale production, the SCs-derived dECM based materials still hold great promise in neural tissue engineering.

## Conclusion and Future Perspectives

SCs are the major glial cells in peripheral nervous system, which provide a permissive substrate for axonal growth, wrap around axons for myelination, and promote functional recovery after nerve injury. Transplantation of SCs has emerged as a promising cell-based therapy for treatments of nervous system diseases, such as long-distance peripheral defects, spinal cord injury, and neurodegenerative disorders. However, a few limitations still need to be overcome, including the insufficient sources of autogenous SCs, and poor cell survival upon injection to the injured area. The SCLCs differentiated from stem cells exhibit great promise as the SCs substitutes for nerve regeneration, but similar problems still exist.

Biomaterials can be used as cell carriers to assist SCs transplantation to the central and peripheral nervous system. These biomaterials, including hydrogels and scaffolds, not only provide cell substrates to avoid damages and anoikis during transplantation, but also can be engineered to promote cell viability, proliferation, and functionalization. Among these SCs transplantation platforms, it is highly desired to build a favorable microenvironment for SCs at both molecular and physiological levels. Additionally, the incorporation and spatial/temporal distributions of the stimulating factors (growth factors, exosomes, and other cells) that manipulate SCs phenotype are also valuable for SCs transplantation.

Although great advancements have been achieved in clinical applications, the cell-based therapy still faces serious limitations, such as the quantity of cells, timing of the treatment, high costs, and potential immune response. Therefore, alternative approaches using the secreta of SCs or SCLCs, such as their secreted exosomes and ECM have been actively developed and shown great promise in facilitating nerve regeneration. However, due to the complexity of exosomes and dECM, the specific roles of each component and the mechanisms on neural regeneration are barely investigated. Future studies focusing on understanding the role of SCs-derived exosomes and dECM can help evaluate the safety and effectiveness of these rapidly advancing biomaterials toward practical use in future clinical applications.

## Author Contributions

YB and DQ provided the idea for the review. ZR searched the literature, prepared the figures, and wrote the manuscript. ZL and PS participated in reviewing the literature. YB contributed to editing and approval of the final manuscript. All authors contributed to the article and approved the submitted version.

## Funding

This work was supported by National Natural Science Foundation of China (51903255, 52073314, and 32171353), the Key Areas Research and Development Program of Guangdong (2020B1111150003), Natural Science Foundation of Guangdong (2022A1515011388), Science and Technology Program of Guangzhou (201904010364), and Science and Technology Projects in Guangzhou (202002020078).

## Conflict of Interest

The authors declare that the research was conducted in the absence of any commercial or financial relationships that could be construed as a potential conflict of interest.

## Publisher's Note

All claims expressed in this article are solely those of the authors and do not necessarily represent those of their affiliated organizations, or those of the publisher, the editors and the reviewers. Any product that may be evaluated in this article, or claim that may be made by its manufacturer, is not guaranteed or endorsed by the publisher.
